# The effects of reduced protein diet supplemented with arginine plus glutamine–glutamate on growth performance, diarrhea incidence, blood metabolites, nutrient digestibility, and nitrogen utilization in nursery pigs

**DOI:** 10.5713/ab.250702

**Published:** 2025-11-10

**Authors:** Lucas Medina Teixeira, Jansller Luiz Genova, Fernanda Fialho Abranches, Gabriel Cipriano Rocha

**Affiliations:** 1 Muscle Biology and Nutrigenomics Laboratory, Department of Animal Science, Universidade Federal de Viçosa, Viçosa, Brazil

**Keywords:** Conditionally Essential Amino Acids, Nitrogen Utilization, Nutrient Digestibility, Protein Reduction, Weaned Piglets

## Abstract

**Objective:**

This study evaluated the effects of reducing crude protein (CP) and supplementing arginine plus glutamine–glutamate (AGG) on growth performance, diarrhea incidence, blood metabolites, amino acid profiles, nutrient digestibility, and nitrogen (N) utilization in nursery pigs.

**Methods:**

A total of 200 entire male and female piglets weaned at 20 d of age (4.8±0.58 kg) were allotted to 5 diets in a randomized block design: 22.5%, 21.0%, 19.5%, or 18.0% CP, and 18.0% CP supplemented with 5 g/kg L-arginine and 10 g/kg L-glutamine+glutamate. Pigs were fed in two phases (20–32 and 32–44 d of age) over a 24-d period.

**Results:**

Reducing dietary CP linearly decreased feed efficiency, with the lowest performance observed in pigs fed the 18.0% CP diet with AGG. Serum urea N and gamma-glutamyl transferase increased linearly with dietary CP level. Plasma arginine and ornithine were elevated by supplementation. Methionine, threonine, and valine were highest in pigs fed the 18.0% CP diets. In contrast, phenylalanine and tyrosine declined with reduced CP. Diarrhea incidence and fecal scores were greater in pigs fed the 22.5% CP, with each 1% CP increase raising diarrhea incidence by 3.55% points. Digestibility of protein and energy improved with increasing dietary CP, and pigs fed 22.5% CP showed the greatest N absorption but also higher fecal N excretion.

**Conclusion:**

Collectively, results indicate that reducing dietary CP from 22.5% to 18.0% compromises growth and nutrient digestibility, even when supplemented with arginine and glutamine–glutamate, but lowers diarrhea incidence and N output. These findings highlight trade-offs between growth efficiency, gut health, and environmental sustainability in nursery pig nutrition.

## INTRODUCTION

Dietary protein is a key determinant of growth and health in weaned piglets, providing both essential amino acids (EAA) and non-essential amino acids (NEAA) required for protein accretion. In addition to their role in protein synthesis, NEAA also contribute to nitrogen (N) metabolism, intestinal development, and immune regulation [1,2]. Conventional nursery diets are often formulated with relatively high levels of crude protein (CP), averaging 20.5%, to secure an adequate amino acid supply [3]. However, excessive CP increases feed costs, N excretion, and the risk of post-weaning diarrhea due to elevated protein fermentation in the hindgut [4,5]. Thus, strategies that allow for a reduction in dietary CP while maintaining piglet performance and health are of increasing interest for both economic and environmental sustainability.

Reducing dietary CP while supplementing crystalline EAA has therefore emerged as a strategy to minimize N excretion and digestive disturbances. Nevertheless, several studies have shown that piglets fed low-CP EAA-fortified diets still exhibit reduced growth and feed efficiency compared to those fed higher-CP diets [6–8]. This reduction in performance is attributed to an inadequate supply of amino acids that become limiting when dietary CP is reduced, including conditionally essential ones such as arginine and glutamine, and non-essential but functionally important amino acids such as glutamate, glycine, and serine, which are more abundant in intact protein sources [1,9].

Among these, arginine, glutamine and glutamate play important roles in intestinal integrity, N disposal, and immune regulation [9,10]. They serve as substrates for proliferating enterocytes and immune cells, precursors for polyamine and nucleotide biosynthesis, modulators of nitric oxide synthesis, and energy sources for epithelial repair, with reported benefits for villus development, barrier function, and N utilization [11–13].

Although such functions suggest that supplementation with selected amino acids such as arginine, glutamine and glutamate could offset limitations of low-CP diets, evidence in nursery pigs remains inconsistent. Previous studies have reported that reducing dietary CP while meeting EAA requirements decreases serum urea N (SUN) and fecal N output, indicating improved N utilization [14,15]. However, crystalline amino acids differ from intact proteins in absorption kinetics, which may alter plasma amino acid patterns and protein synthesis efficiency [9,16]. Moreover, dietary CP level and protein source affect energy and nutrient digestibility [17]. High-CP diets, in contrast, may increase hepatic metabolic load, as suggested by serum gamma-glutamyl transferase (GGT) activity [18]. Yet, the extent to which supplementation with arginine plus glutamine-glutamate (AGG) can compensate for reduced dietary CP levels has not been systematically investigated in nursery piglet diets.

Based on this background, we hypothesized that reducing dietary CP levels, when associated with AGG supplementation, would not impair growth performance, diarrhea incidence, blood profile, or nutrient digestibility in nursery pigs. Therefore, the objective of this study was to evaluate the effect of dietary CP levels and AGG supplementation on growth performance, blood profile, diarrhea incidence, apparent digestibility of energy and protein, and N utilization and fecal losses in nursery pigs.

## MATERIALS AND METHODS

### Animals, diets, experimental design, and housing

A total of 200 piglets (PIC 337 [Large White×Landrace×Duroc ×Pietrain]×DB 90 [Large White×Landrace]) entire males and females, weaned at 20 d of age and with 4.80±0.58 kg body weight (BW) were allotted in a randomized complete block design based on initial BW to one of five dietary treatments and eight replicates. Piglets were not creep-fed during the suckling period. The treatments consisted of diets containing 22.5%, 21.0%, 19.5%, or 18.0% CP and a fifth treatment with 18.0% CP supplemented with 5 g/kg L-arginine (purity> 99.0%) and 10 g/kg L-glutamine+L-glutamate (minimum 10% L-glutamine and 10% L-glutamate). Pigs were fed according to a two-phase nursery feeding program: phase 1 from 20 to 32 d of age and phase 2 from 32 to 44 d of age. All diets were formulated to meet the nutritional recommendations of the Brazilian Tables for Poultry and Swine ([19]; Table 1) and were provided in mash form.

The 24-d experiment was conducted in a commercial barn in the municipality of Santo Antônio do Grama, Minas Gerais, Brazil. The pigs were housed in suspended pens (1.75× 1.00 m), with five piglets per pen (0.35 m^2^/pig), and had free access to feed and water. The minimum and maximum temperatures in the nursery room were 18.7±1.55°C and 30.9± 1.77°C, respectively.

### Performance and diarrhea incidence

Throughout the trial, the offered diet and leftovers were weighed to calculate average daily feed intake (ADFI). Pigs were individually weighed on d 20, 32, and 44 of age to estimate BW, average daily weight gain (ADG) and gain-to-feed ratio (G:F).

The fecal consistency of each pig was visually assessed from 8:00 to 10:00 h during phase 1 and phase 2, using the method described by Liu et al [20]. Fresh feces were ranked on a 4-point scale as follows: 0 = solid, 1 = semi-solid, 2 = semi-liquid, and 3 = liquid. Diarrhea incidence was defined as the consistency of feces at scale 2 or 3 for 2 consecutive days. Diarrhea incidence per pen (%) was calculated as follows: (number of animals with diarrhea in each pen × day of diarrhea) ÷ (total number of animals in the pen × day observed) ×100.

### Blood collection and analysis

At 44 d of age, blood was collected from 1 piglet per pen, selected by BW closest to the pen average; pigs were not fasted before sampling. Blood was collected at 7:00 a.m. by orbital sinus puncture using a hypodermic needle (40×1.6 mm) into 2 tubes of 9 mL each, one containing anticoagulant (sodium heparin) and one without. Then, blood was centrifuged at 4,000×g for 12 min to separate serum or plasma. Serum and plasma samples were sent to the Viçosa Clinical Laboratory. Serum concentrations of SUN (Ureal Cobas C311, Linklab, PNCQ software), creatinine (WS-Kovalent, kinetic method, BS-380, Mindray), immunoglobulin G (IgG; Atellica CH IgG_2, CH Analyzer; Siemens Healthineers), GGT (GGT2 IFCC, kinetic colorimetric method, Cobas C311 analyzer; Roche Diagnostics), aspartate aminotransferase (AST; ASTL IFCC, kinetic UV method with pyridoxal phosphate activation, Cobas C311 analyzer; Roche Diagnostics) and alanine aminotransferase (ALT; ALTL IFCC, kinetic UV method with pyridoxal phosphate activation, Cobas C311 analyzer; Roche Diagnostics) were determined using commercial kits, according to the manufacturer’s instructions. In plasma samples, the amino acid profile was evaluated by liquid chromatography coupled with tandem mass spectrometry (LC-MS/MS).

### Apparent total tract digestibility of energy and protein, and nitrogen utilization and fecal losses

Feed samples (100 g) from each of the five treatments were collected at the beginning of the experimental phase 2. Fecal samples were collected from each pen, totaling 200 g per pen as the combined amount from d 43 to 44. During the collection, feces were stored in identified polyethylene plastic bags and kept in cooled thermal boxes (4°C). All fecal and feed samples were stored in a refrigerator at −20°C. Subsequently, fecal samples from each pen were thawed at room temperature for 4 h, homogenized and dried in a forced-air oven at 55°C for 72 h. After drying, both fecal and feed samples were ground using a ball mill and stored in polyethylene jars for dry matter (DM), CP (N×6.25), gross energy (GE), and titanium dioxide (TiO_2_) contents analyses.

Apparent digestibility was determined using the indicator method with TiO_2_, which was included in the phase 2 diet at a content of 4 g/kg, 4 d before collection began [21]. For the digestibility to determine the TiO_2_ contents in feces and diet analysis, the procedure used was digestion with sulfuric acid (Method M-007/2), as described by Detmann et al [22]. The GE content of the diets and feces was measured using an adiabatic calorimeter bomb (Model 1356; Parr Instrument Company). The N content was determined by the Kjeldahl method (Method N-001/2). N intake and excretion were calculated by multiplying the respective N contents of feed and feces by feed intake and fecal output, respectively.

The apparent digestibility coefficients (ADC) of GE, CP and N were calculated according to the equations of Adeola [23].


(1) ADC (%)=100-(100×{[Mfeed×Cfeces]/[Mfeces×Cfeed]}),

where: Mfeed and Mfeces is the content of TiO_2_ in the feed and feces, and Cfeed and Cfeces is the content of the nutrient or energy in the feed and feces, respectively.

From these coefficients, the values of digestible protein (DP) and energy (DE) were obtained by multiplying the respective coefficients to the dietary contents of CP or GE, with DP expressed as percentage of the diet and DE expressed in kcal/kg of diet.

### Statistical analysis

Prior to statistical analyses, data were examined for potential outliers by inspecting studentized residuals in the Proc Univariate procedure. Observations with residuals exceeding ±3 standard deviations from the mean were considered outliers and removed. The normality of residuals was verified using the Shapiro–Wilk test. Data were analyzed by analysis of variance (ANOVA) using SAS 9.4 software (SAS Institute), with treatments considered as fixed effects and block (e.g., initial BW) as a random effect in the Proc Mixed procedure. When a significant difference was detected by the F-test in the type III analysis, least squares means were compared using Tukey’s post hoc test at a 5% significance level. In addition, responses to dietary CP levels were evaluated using linear and quadratic regression models in the Proc Reg procedure, with the significance of the coefficients tested by the t-test. When a quadratic effect was observed, the maximum or minimum critical point was determined based on the derivative of the second-degree polynomial. In all analyses, differences were considered significant at p<0.05.

## RESULTS

### Performance and diarrhea incidence

Reducing CP levels from 22.5% to 18.0%—with or without supplementation of AGG—significantly affected the performance of piglets between 21 and 44 d of age. G:F decreased (p<0.05 for phase 1, phase 2 and the overall period) in pigs fed the 18.0% CP diet supplemented with AGG compared to the 22.5% CP diet (Table 2). The linear regression revealed that for each 1.0% increase in CP, G:F increased by 0.018 units during phase 1, by 0.019 during phase 2, and by 0.015 over the total period. Average daily gain and final BW were also reduced (p<0.05 for phase 2 and the overall period), with the lowest values observed in the 18.0% CP supplemented with AGG compared to the group than 22.5% CP group.

Regarding diarrhea results, diarrhea incidence and scores were highest (p<0.05) in piglets fed 22.5% CP diet than those fed 19.5%, 18.0% or 18.0% CP diets supplemented with AGG for both phases and overall period (Table 3). Regression analysis indicated that each 1.0% increase in CP raised diarrhea incidence by 3.55% points and increased the diarrhea score by 0.111 in the overall period.

### Biochemical and immunological blood profile

For serum metabolites, SUN was higher (p<0.05) in pigs with the 22.5% CP diet compared to the 19.5% or 18.0% CP diets, and increased by 0.704 mg/dL for every 1.0% increase in CP (Table 4). Gamma glutamyl transferase also showed a linear increase of 0.042 μkat/L per 1.0% increase in CP, despite no pairwise differences detected by Tukey’s test. However, there was no treatment effect on creatinine, AST, ALT, and IgG concentrations.

### Amino acid blood profile

In the amino acid blood profile, Tukey’s test revealed that Arg, Phe, Met, Orn, Tyr, Thr, and Val were affected (p<0.05) by CP levels (Table 5). For Arg, piglets receiving the 18.0% CP diet supplemented with AGG had higher concentrations than those fed 18.0% CP, with the 18.0% CP group exhibiting the lowest values. For Phe, the highest concentrations were observed in the 22.5% and 21.0% CP groups compared to the 18.0% CP+AGG group. Methionine concentrations were higher in the 18.0% and 18.0% CP+AGG groups than the 22.5% and 21.0% groups. For Orn, the 18.0% CP+AGG group had higher concentrations compared to the 21.0%, 19.5%, and 18.0% CP groups. Regarding Tyr, the highest levels were observed with the 22.5% and 21.0% CP diets in comparison with 18.0% CP+AGG group. For Thr, concentrations were highest in the 18.0% group, which differed from the 22.5% group. For Val, both the 18.0% and 18.0% CP+AGG groups had higher concentrations than 22.5% and 21.0% CP groups. Arginine concentration increased by 6.499 μmol/L for each 1.0% rise in CP, while Met and Val concentrations decreased by 5.985 and 11.602 μmol/L, respectively, for every 1.0% increase in CP. The quadratic model for Glu showed a critical minimum at 20.5% CP, while Orn, Ser, and Val reached a minimum at 19.2%, 19.9% and 23.9% CP, respectively.

### Apparent total tract digestibility of energy and protein, and nitrogen utilization and fecal losses

Piglets fed the 22.5% CP showed (p<0.05) the highest DE, which differed from all other groups (Table 6). The 21.0% group had the second highest value, higher than the 19.5% and 18.0% CP diets supplemented with arginine and glutamine–glutamate, and also higher than the 18.0% CP group. For the ATTDGE, the 22.5% CP group was higher (p<0.05) than the 21.0% and 18.0% CP groups, which in turn were higher than the 19.5% and 18.0% CP+AGG groups. The lowest value was observed for the 18.0% CP+AGG group, which was lower than all others. For DP, the highest value occurred in pigs fed the 22.5% CP diet, which was significantly greater than the 21.0%, 19.5%, 18.0%, and 18.0% CP+AGG diets, with all diets differing from each other. For the ATTDCP, the 22.5% CP group was higher (p<0.05) than the 21.0% CP and both were higher than the 19.5% and 18.0% CP groups. The 18.0%+AGG group did not differ statistically from the 21.0% or 18.0% CP groups. N intake was higher (p<0.05) in pigs with the 22.5% CP diet, which differed from the 21.0% and 18.0% CP groups, with the 19.5% and 18.0%+AGG diets showing intermediate results. Regarding N in feces, the 22.5% CP group excreted (p<0.05) more N than the 18.0% group, with the other groups being statistically intermediate and not differing from those with 18.0% CP. Piglets fed diets containing 22.5% CP showed higher N absorption than the other CP levels (p<0.05). The apparent digestibility of N was higher (p<0.05) in the 22.5% CP group than in the 21.0% and 18.0% CP groups, with the 19.5% and 18.0% CP+AGG groups showing intermediate results. For DP, each 1.0% increase in CP raised digestibility by 1.069% points, and N excretion in feces increased by 0.889 g per 1.0% CP increment.

## DISCUSSION

The reduction of dietary CP from 22.5% to 18.0% resulted in a decrease in ADG (phase 2) and G:F of weaned piglets, indicating that the supplementation of EAA alone was insufficient to sustain optimal growth performance under lower dietary CP conditions. These findings are consistent with previous reports showing impaired growth in pigs fed reduced-protein diets despite meeting EAA requirements [7,8,24]. The decline in performance may be explained by an inadequate supply of amino acids that become limiting when dietary protein is reduced, particularly those with functional roles such as arginine, glutamine, and glutamate [1,3,9]. Because arginine is considered essential for young pigs rather than non-essential, its inclusion together with glutamine–glutamate represents a targeted functional supplementation rather than NEAA addition. However, in the present study, supplementation with arginine and glutamine/glutamate did not restore performance to the level observed with the highest CP diet, suggesting that the synergistic effect of these amino acids was insufficient to overcome the reduced supply of other nitrogenous compounds from intact protein [25].

In our experimental design, the arginine supplementation was intended to restore the SID arginine level to the recommended requirement for nursery pigs (approximately 1.35%; Rostagno et al [19]). Therefore, an improvement in growth performance was expected, consistent with our initial hypothesis. However, this effect was not confirmed, indicating that other limiting factors—possibly related to the availability of non-protein N sources or the functional role of glutamine–glutamate—may have constrained the response. Furthermore, the better performance of piglets fed the 22.5% CP diet could also be related to the improved digestibility of nutrients observed at higher protein levels, as intact proteins contribute to a more balanced amino acid profile and efficient utilization of N.

This interpretation is further supported when the results are considered in light of the dietary SID Lys:CP ratios. In our experiment, piglets fed 18.0% CP diets had ratios of 8.05% (phase 1) and 7.50% (phase 2), both above the thresholds suggested in previous studies. Millet et al [7] reported that when the SID Lys:CP ratio exceeds approximately 6.4%, CP rather than lysine becomes limiting for protein deposition, impairing growth performance. Similarly, Rocha et al [3] estimated that 6.6% SID Lys:CP represents the breakpoint above which both ADG and G:F are compromised. Consistently, piglets fed 22.5% CP diets in our study had SID Lys:CP ratios of 6.44% and 6.00%, values much closer to these reported optimal ranges, which supports their superior G:F and ADG. Taken together, these findings reinforce the concept that very high SID Lys:CP ratios (>7%) may indicate insufficient N or NEAA supply, thereby limiting protein accretion even under adequate lysine provision.

Interestingly, although piglets fed the lowest CP diets showed reduced growth performance, they also exhibited a marked reduction in diarrhea incidence. This agrees with earlier findings that lower dietary protein decreases the amount of undigested protein reaching the hindgut, thereby reducing substrate availability for pathogenic bacteria and fermentation that can lead to diarrhea [4,5,15]. Thus, while higher CP diets improved growth rate, they simultaneously increased the risk of digestive disturbances, highlighting a trade-off between maximizing performance and maintaining gut health. Although AGG supplementation did not reduce diarrhea incidence in this study, arginine, glutamine, and glutamate are known to support intestinal integrity and immune function [9,10], which could explain why their use is still considered beneficial in nursery nutrition. These findings reinforce the complexity of balancing dietary protein levels in nursery pigs, where both growth efficiency and gastrointestinal health must be considered.

SUN concentrations decreased linearly with the reduction of dietary CP, reflecting a lower N intake and improved efficiency of N utilization under reduced-protein conditions. This response is in agreement with Figueroa et al [26] and Heo et al [14], who demonstrated that lowering CP while meeting EAA requirements reduces N excretion and blood urea levels. The reduction in SUN indicates that piglets on low-CP diets catabolized less amino acid N for energy, which is consistent with the environmental benefits of protein reduction strategies. The linear increase in GGT in piglets fed high-CP diets may reflect a greater hepatic metabolic load associated with the increased deamination of amino acids and N detoxification, as GGT plays a role in amino acid transport and glutathione turnover [18,27]. In contrast, ALT and AST remained unchanged, indicating the absence of overt hepatocellular damage. This pattern supports the interpretation that high-protein diets impose a subclinical metabolic burden on the liver, reflected by GGT leakage into the bloodstream, rather than causing acute hepatic damage [28,29]. Therefore, GGT appears to be a sensitive but not exclusive biomarker of hepatic stress and response to dietary protein level, and its interpretation should be considered alongside other metabolic indicators.

Plasma amino acid profiles were markedly influenced by dietary CP level and the addition of AGG. Supplementation with AGG increased circulating arginine and ornithine. In parallel, a linear rise in plasma concentrations of both amino acids was also detected with increasing CP levels, corroborating the elevated SUN values in piglets receiving high-protein diets. Arginine and ornithine serve as crucial intermediates in activating the urea cycle, thereby enhancing ammonia detoxification and maintaining N homeostasis [30]. These results suggest that both protein level and targeted supplementation with functional amino acids modulate the availability of amino acids involved in N disposal, providing a mechanistic link between dietary CP supply, circulating amino acid patterns, and serum urea responses.

Plasma methionine, threonine, and valine concentrations were higher in piglets receiving low-CP diets. This response is likely a direct consequence of the increased inclusion of crystalline amino acids in these formulations. Unlike amino acids bound to intact proteins, crystalline forms are nearly 100% digestible and absorbed more rapidly in the small intestine [9,12], which favors their appearance in circulation. Crystalline amino acids are absorbed rapidly and almost completely in the small intestine, whereas amino acids from intact proteins are released more gradually during digestion, leading to temporal asynchrony between EAA and NEAA [16]. Such asynchrony can reduce their efficiency for protein synthesis [31–33], helping to explain why piglets on low-CP diets showed poorer growth performance despite elevated plasma concentrations. At the same time, the lower supply of intact protein reduced the amount of undigested N reaching the hindgut, which likely contributed to the reduced incidence of diarrhea [4,5]. Thus, the combination of metabolic inefficiency due to amino acid imbalance and improved gut health reflects the trade-off observed when formulating low-CP diets fortified with crystalline amino acids.

In contrast, aromatic amino acids such as phenylalanine and tyrosine declined with reduced CP, reflecting their predominant contribution from intact protein sources. Because these amino acids are essential precursors for hormones and neurotransmitters and also contribute to protein synthesis [34,35], their lower circulating concentrations may represent an important nutritional limitation of low-CP diets. This imbalance could partially explain the reduced growth performance of piglets under protein restriction, despite the elevated concentrations of other indispensable amino acids.

Energy and protein digestibility were significantly influenced by dietary CP level, with piglets fed high-CP diets exhibiting superior values compared to those on reduced-CP treatments. These findings are consistent with previous research showing that excessively low CP levels, even when balanced for EAA, can compromise pig performance [3,7,15]. The linear decline in G:F observed in our study reinforces this conclusion, indicating that the nutrient density and amino acid balance of the low-CP diets were insufficient to sustain optimal efficiency. Beyond amino acid adequacy, our results may also be affected by the energy values attributed to SBM in feed formulation systems. In our diets, SBM was assigned a metabolizable energy value of 3,240 kcal/kg according to Rostagno et al [19]; however, emerging evidence suggests that the metabolizable or productive energy of SBM may be underestimated in standard nutrient tables [17,36,37]. This underestimation could help explain why diets with greater proportions of SBM not only improved protein supply but also yielded superior energy digestibility and growth performance. Consequently, the decline in energy digestibility in the low-CP treatments may not solely reflect amino acid limitations or the absence of bioactive compounds from intact proteins, but also the reduced contribution of SBM’s true energy value when it was replaced with other ingredients. However, this hypothesis needs to be further investigated.

Despite the observed decline in piglet performance and nutrient digestibility, the reduction in fecal N at lower CP levels holds significant environmental relevance. The linear decrease in N intake observed in this study directly translates into lower fecal N excretion. This is a critical point, as a primary environmental concern associated with swine production is the excessive release of reactive N compounds into the atmosphere and soil [38]. Therefore, from a sustainability perspective, the practice of reducing dietary CP, even when it compromises some performance metrics, is a crucial strategy for mitigating the environmental footprint of swine production. This showed clear trade-off between maximizing production efficiency and minimizing ecological impact, highlighting the need for a more holistic approach to diet formulation that considers both animal performance and environmental stewardship.

It may also be argued that growth depression resulting from a low-protein diet during the nursery stage could have long-term consequences, limiting compensatory growth during the grow-finish period. Conversely, low-protein strategies applied later in production can be economically and environmentally advantageous, as they reduce N excretion without compromising carcass quality. Therefore, while the 18% CP diet with AGG supplementation was not effective in sustaining growth performance in nursery pigs, such a concept could be further explored for the grow-finish stage, where amino acid requirements are lower and protein efficiency becomes more relevant to sustainability goals.

## CONCLUSION

Reducing CP from 22.5% to 18.0%, even with AGG supplementation, decreased average daily gain, feed efficiency, and nutrient digestibility in nursery pigs. However, lower protein diets reduced diarrhea incidence and N excretion. These results indicate that while high-CP diets improve growth performance and N utilization efficiency, protein reduction offers benefits for intestinal health and lowering environmental N output. Nursery pigs with 18% CP and AGG feeding have environmental benefits but not production value.

## Figures and Tables

**Table 1:** Ingredients and chemical composition of diets fed to nursery pigs (as-fed basis)

Ingredients (g/kg)	Phase 1 (21 to 32 d of age)					Phase 2 (32 to 44 d of age)				
	
	22.5%	21.0%	19.5%	18.0%	18.0%+AGG	22.5%	21.0%	19.5%	18.0%	18.0%+AGG
Ground corn (7.8% CP)	390.0	433.6	479.2	526.5	526.5	423.0	465.4	509.0	554.7	554.7
Soybean meal (46.0% CP)	227.1	181.0	131.8	80.3	80.3	293.7	249.4	203.3	153.9	153.9
Dried whey (12.5% CP)	150.0	150.0	150.0	150.0	150.0	100.0	100.0	100.0	100.0	100.0
Soybean micronized (36.0% CP)	100.0	100.0	100.0	100.0	100.0	70.0	70.0	70.0	70.0	70.0
Plasma protein (78.0% CP)	40.0	40.0	40.0	40.0	40.0	20.0	20.0	20.0	20.0	20.0
Sugar	30.0	30.0	30.0	30.0	30.0	30.0	30.0	30.0	30.0	30.0
Corn starch	15.0	15.0	15.0	15.0	0.0	15.0	15.0	15.0	15.0	0.0
AGG1)	0.0	0.0	0.0	0.0	15.0	0.0	0.0	0.0	0.0	15.0
Bicalcium phosphate	12.0	12.4	12.8	13.2	13.2	12.9	13.3	13.7	14.1	14.1
Limestone calcitic	8.6	8.8	8.9	9.1	9.1	8.1	8.3	8.4	8.6	8.6
Soybean oil	10.5	8.7	6.4	3.6	3.6	15.1	13.6	11.8	9.5	9.5
Salt (NaCl)	3.4	3.4	3.4	3.4	3.4	1.7	1.7	1.7	1.7	1.7
L-lys (78.0%)	3.0	4.4	5.9	7.5	7.5	2.3	3.6	5.0	6.5	6.5
DL-met (99.0%)	1.8	2.2	2.7	3.1	3.1	1.3	1.7	2.2	2.6	2.6
L-thr (98.5%)	1.5	2.1	2.8	3.5	3.5	1.1	1.8	2.4	3.1	3.1
L-val (96.5%)	0.1	0.9	1.8	2.7	2.7	0.0	0.3	1.1	2.0	2.0
L-trp (99.0%)	0.1	0.3	0.6	0.9	0.9	0.0	0.2	0.4	0.7	0.7
L-ile (98.0%)	0.0	0.2	1.1	2.1	2.1	0.0	0.0	0.3	1.2	1.2
L-his (98.0%)	0.0	0.0	0.5	1.2	1.2	0.0	0.0	0.0	0.6	0.6
L-leu (99.5%)	0.0	0.0	0.0	0.9	0.9	0.0	0.0	0.0	0.0	0.0
Zinc oxide	3.0	3.0	3.0	3.0	3.0	2.2	2.2	2.2	2.2	2.2
Choline chloride	2.0	2.0	2.0	2.0	2.0	1.5	1.5	1.5	1.5	1.5
Vitamin-mineral premix2)	1.4	1.4	1.4	1.4	1.4	1.4	1.4	1.4	1.4	1.4
Copper sulfate	0.6	0.6	0.6	0.6	0.6	0.6	0.6	0.6	0.6	0.6
Calculated composition (%)3)										
ME (kcal/kg)	3,400	3,400	3,400	3,400	3,400	3,375	3,375	3,375	3,375	3,375
CP	22.5	21.0	19.5	18.0	19.5	22.5	21.0	19.5	18.0	19.5
Standardized ileal digestible lys	1.45	1.45	1.45	1.45	1.45	1.35	1.35	1.35	1.35	1.35
Standardized ileal digestible met	0.45	0.47	0.49	0.52	0.52	0.41	0.43	0.46	0.48	0.48
Standardized ileal digestible met+cys	0.81	0.81	0.81	0.81	0.81	0.75	0.75	0.75	0.75	0.75
Standardized ileal digestible thr	0.97	0.97	0.97	0.97	0.97	0.9	0.9	0.90	0.90	0.90
Standardized ileal digestible trp	0.28	0.28	0.28	0.28	0.28	0.27	0.26	0.26	0.26	0.26
Standardized ileal digestible val	1.00	1.00	1.00	1.00	1.00	0.97	0.93	0.93	0.93	0.93
Standardized ileal digestible arg	1.30	1.16	1.01	0.86	1.33	1.35	1.22	1.10	0.94	1.41
Standardized ileal digestible ile	0.85	0.80	0.80	0.80	0.80	0.87	0.79	0.74	0.74	0.74
Total Ca	0.85	0.85	0.85	0.85	0.85	0.83	0.83	0.83	0.83	0.83
Standardized total tract digestible P	0.50	0.51	0.51	0.51	0.51	0.47	0.47	0.47	0.47	0.47
Total Na	0.40	0.40	0.40	0.40	0.40	0.23	0.23	0.23	0.23	0.23
Lactose	11.25	11.25	11.25	11.25	11.25	7.50	7.50	7.50	7.50	7.50

1) AGG, 5 g/kg arg (L-arginine; purity>99.0%), 10 g/kg glu+gln (min 10.0% L-glutamine+min 10.0% L-glutamate).

2) Provided the following quantities per kg of complete diet: copper sulfate, 10 mg; potassium iodate, 1.5 mg; iron sulfate, 100 mg; manganese sulfate, 40 mg; sodium selenite, 0.3 mg; zinc oxide, 100 mg; vitamin A, 12,000 IU; vitamin D_3_, 2,250 IU; vitamin E, 65 IU; vitamin K_3_, 3 mg; thiamine, 2.25; riboflavin, 6 mg; pyridoxine, 2.25 mg; vitamin B_12_, 27 mcg; folic acid, 400 mcg; biotin, 150 mcg; pantothenic acid, 22.5 mg; niacin, 45 mg.

3) Calculated composition of dietary ingredients based on the Brazilian Tables [19].

CP, crude protein.

**Table 2:** Effect of crude protein (CP) levels and arginine+glutamine–glutamate (AGG) on nursery pig growth performance

Item	Levels of CP (%)					SEM1)	p-value2)	Regression3)	
	
	22.5	21.0	19.5	18.0	18.0+AGG			Lin	Quad
Initial BW (kg)	4.8	4.8	4.8	4.8	4.8	0.093	-	-	-
Phase 1 (20 to 32 d of age)									
Final BW (kg)	7.11	6.92	7.02	6.87	6.69	0.112	0.336	0.259	0.920
ADG (kg)	0.19	0.18	0.19	0.17	0.16	0.004	0.110	0.326	0.732
ADFI (kg)	0.27	0.25	0.27	0.28	0.25	0.005	0.289	0.487	0.408
G:F (kg:kg)4)	0.71^a^	0.70^a^	0.67^ab^	0.63^b^	0.63^b^	0.073	0.001	0.030	0.094
Phase 2 (32 to 44 d of age)									
Final BW (kg)	11.45^a^	11.12^ab^	11.21^ab^	10.81^ab^	10.59^b^	0.159	0.017	0.107	0.892
ADG (kg)	0.36^a^	0.35^ab^	0.35^ab^	0.33^b^	0.32^b^	0.005	0.007	0.077	0.592
ADFI (kg)	0.54	0.54	0.55	0.56	0.52	0.007	0.176	0.056	0.268
G:F (kg:kg)4)	0.68^a^	0.66^ab^	0.64^ab^	0.59^c^	0.62^bc^	0.051	<0.001	0.030	0.268
Overall (20 to 44 d of age)									
ADG (kg)	0.28^a^	0.26^ab^	0.27^ab^	0.25^ab^	0.24^b^	0.004	0.009	0.200	1.000
ADFI (kg)	0.40	0.39	0.41	0.40	0.39	0.005	0.374	0.684	1.000
G:F (kg:kg)4)	0.69^a^	0.67^ab^	0.65abc	0.62^c^	0.63^bc^	0.048	<0.001	0.005	0.268

1) Pooled standard error of the mean.

2) Refer to ANOVA.

3) Refer to t-tests for each regression coefficient.

4) 20 to 32 d: G:F = 0.313+0.018×CP, R^2^ = 0.94; 32 to 44 d: G:F = 0.251+0.019×CP, R^2^ = 0.94; 0 to 44 d: G:F = 0.347+0.015×CP, R^2^ = 0.99.

a–c Statistically different by Tukey’s post hoc test (p<0.05).

BW, body weight; ADG, average daily gain; ADFI, average daily feed intake; G:F, gain to feed ratio; ANOVA, analysis of variance.

**Table 3:** Effect of crude protein (CP) levels and arginine+glutamine–glutamate (AGG) on the fecal score and diarrhea incidence of nursery pigs

Item	Levels of CP (%)					SEM1)	p-value2)	Regression3)	
	
	22.5	21.0	19.5	18.0	18.0+AGG			Lin	Quad
Phase 1 (20 to 32 d of age)									
Fecal score4)	0.83^a^	0.73^a^	0.36^b^	0.36^b^	0.37^b^	0.051	<0.001	0.063	0.763
Diarrhea incidence (%)5)	25.25^a^	22.75^a^	10.75^b^	10.63^b^	8.00^b^	1.858	<0.001	0.070	0.845
Phase 2 (20 to 32 d of age)									
Fecal score	0.64^a^	0.25^b^	0.23^b^	0.14^b^	0.10^b^	0.050	0.001	0.116	0.370
Diarrhea incidence (%)	17.25^a^	3.00^b^	2.50^b^	0.50^b^	1.00^b^	1.729	0.006	0.149	0.323
Overall (20 to 44 d of age)									
Fecal score6)	0.74^a^	0.49^ab^	0.29^b^	0.25^b^	0.23^b^	0.044	<0.001	0.039	0.146
Diarrhea incidence (%)6)	21.25^a^	12.88^ab^	6.62^b^	5.56^b^	4.50^b^	1.486	<0.001	0.045	0.119

1) Pooled standard error of the mean.

2) Refer to ANOVA.

3) Refer to t-tests for each regression coefficient.

4) Fecal consistency: 0 = solid; 1 = semi-solid; 2 = semi-liquid; and 3 = liquid.

5) Diarrhea incidence was considered to be the presence of feces with a score of 2 or 3 for two consecutive d.

6) FS =−1.812+0.111×CP, R^2^ = 0.92; DI = −60.418+3.555× CP, R^2^ = 0.91.

a,b Statistically different by Tukey’s post hoc test (p<0.05).

ANOVA, analysis of variance.

**Table 4:** Effect of crude protein (CP) levels and arginine+glutamine–glutamate (AGG) on the biochemical and immunological blood profile of nursery pigs with 44 d of age

Item	Levels of CP (%)					SEM1)	p-value2)	Regression3)	
	
	22.5	21.0	19.5	18.0	18.0+AGG			Lin	Quad
SUN (mmol/L)4)	8.75^a^	7.67^ab^	6.29^b^	5.67^b^	6.74^ab^	0.225	0.005	0.009	0.430
Creatinine (μmol/L)	77.49	71.60	68.95	70.72	68.07	0.884	0.411	0.320	0.514
AST (μkat/L)	1.20	1.35	1.12	1.04	1.15	0.057	0.546	0.307	0.510
ALT (μkat/L)	0.96	0.99	0.99	0.99	1.15	0.035	0.408	0.240	0.348
GGT (μkat/L)5)	0.89	0.83	0.75	0.71	0.01	2.149	0.282	0.008	0.582
IgG (g/L)	1.82	1.72	1.74	1.50	1.62	0.060	0.531	0.104	0.579

1) Pooled standard error of the mean.

2) Refer to ANOVA.

3) Refer to t-tests for each regression coefficient.

4) SUN = −7.242+0.708×CP, R^2^ = 0.98

5) GGT = −0.041+0.0413×CP, R^2^ = 0.98.

a,b Statistically different by Tukey’s post hoc test (p<0.05).

SUN, serum urea nitrogen; AST, aspartate aminotransferase; ALT, alanine aminotransferase; GGT, gamma glutamyl transferase; IgG, immunoglobulin G; ANOVA, analysis of variance.

**Table 5:** Effect of crude protein (CP) levels and arginine+glutamine–glutamate (AGG) on the amino acid blood profile (μmol/L) of piglets with 44 d of age

Item	Levels of CP (%)					SEM1)	p-value2)	Regression3)	
	
	22.5	21.0	19.5	18.0	18.0+AGG			Lin	Quad
Argininosuccinic acid	0.41	0.29	0.24	0.21	0.34	0.024	0.053	0.135	0.131
Aspartic acid	30.28	27.40	26.20	27.66	28.09	1.175	0.880	0.320	0.065
Glutamic acid4)	159.90	139.59	143.94	172.25	179.63	6.951	0.310	0.644	0.004
Alanine	278.44	264.16	251.45	321.86	375.29	14.938	0.051	0.505	0.259
Arginine4)	158.15^ab^	145.71^ab^	138.26^ab^	128.14^b^	185.36^a^	6.238	0.034	0.005	0.621
Citrulline	39.85	41.66	39.93	41.06	42.94	1.097	0.909	0.725	0.854
Phenylalanine4)	57.99^a^	57.74^a^	46.01^ab^	40.51^ab^	32.26^b^	2.439	0.001	0.051	0.627
Glycine	503.03	513.71	525.43	631.98	482.68	18.157	0.076	0.138	0.263
Glutamine+lysine	60.53	56.85	59.75	63.40	68.09	2.260	0.611	0.447	0.217
Histidine	73.24	52.18	58.53	60.78	73.04	7.701	0.896	0.546	0.346
Leucine+isoleucine	128.64	124.36	119.10	126.73	108.64	3.937	0.530	0.655	0.306
Methionine4)	45.35^b^	47.78^b^	59.24^ab^	71.45^a^	75.51^a^	2.919	<0.001	0.032	0.231
Ornithine4)	72.78^ab^	62.10^b^	58.55^b^	60.24^b^	79.73^a^	2.652	0.035	0.171	0.043
Proline+aspartic acid	310.15	316.56	290.80	312.43	294.68	11.272	0.944	0.786	0.743
Serine	62.71	55.80	54.40	59.70	46.94	1.935	0.076	0.643	0.028
Tyrosine	60.20^a^	59.19^a^	58.33^ab^	48.03^ab^	35.91^b^	2.849	0.019	0.147	0.275
Threonine	55.88^c^	76.25^bc^	77.79^bc^	124.58^a^	93.31^ab^	5.200	<0.001	0.078	0.526
Tryptophan	45.71	40.90	39.75	49.20	47.16	2.139	0.608	0.725	0.136
Valine	100.55^b^	110.70^b^	128.53^ab^	152.63^a^	158.70^a^	5.000	<0.001	0.016	0.028

1) Pooled standard error of the mean.

2) Refer to ANOVA.

3) Refer to t-tests for each regression coefficient.

4) Glu = 2,409.865–221.55×CP+5.402×CP^2^, R^2^ = 1.00; Arg = 10.967+6.499×CP, R^2^ = 0.99; Phe = −36.067+4.278×CP, R^2^ = 0.90; Met = 177.142–5.985× CP, R^2^ = 0.94; Orn = 567.580–52.920×CP+1.374×CP^2^, R^2^ = 1.00; Ser = 596.574–54.250×CP+1.357×CP^2^, R^2^ = 1.00; Val = 358.038–11.602×CP, R^2^ = 0.97 and Val = 989.275–74.377×CP + 1.550×CP^2^, R^2^ = 1.00.

a–c Statistically different by Tukey’s post hoc test (p<0.05).

ANOVA, analysis of variance.

**Table 6:** Effect of crude protein (CP) levels and arginine+glutamine–glutamate (AGG) on the apparent total tract digestibility of energy and protein, and nitrogen utilization and fecal losses of piglets from 32 to 44 d of age

Item	Levels of CP (%)					SEM1)	p-value2)	Regression3)	
	
	22.5	21.0	19.5	18.0	18.0+AGG			Lin	Quad
DE (kcal/kg)	3,667.9^a^	3,574.1^b^	3,478.3^d^	3,500.0^c^	3,444.3^d^	14.939	<0.001	0.124	0.271
ADCGE (%)	81.54^a^	80.93^b^	79.51^c^	80.63^b^	78.90^d^	0.161	<0.001	0.370	0.454
DP (%)4)	16.73^a^	14.07^b^	12.65^d^	11.86^e^	13.20^c^	0.272	<0.001	0.033	0.092
ADCCP (%)	75.96^a^	73.16^b^	70.09^c^	70.81^c^	70.86^bc^	0.419	<0.001	0.098	0.303
N intake (g)	226.75^a^	193.16^b^	189.21^bc^	173.98^c^	185.63^bc^	3.684	<0.001	0.058	0.499
N in feces (g)5)	48.17^a^	46.94^ab^	45.25^ab^	44.30^b^	44.84^ab^	0.453	0.033	0.005	0.701
N absorbed (g)	178.57^a^	146.22^b^	143.96^b^	129.68^b^	140.78^b^	4.124	<0.001	0.068	0.513
ADCN (%)	78.72^a^	75.53^b^	75.80^ab^	74.40^b^	75.71^ab^	0.444	0.004	0.110	0.578

1) Pooled standard error of the mean.

2) Refer to ANOVA.

3) Refer to t-tests for each regression coefficient.

4) DP = −7.813+1.069×CP, R^2^ = 0.93.

5) Fecal N = 28.167+0.889×CP, R^2^ = 0.99.

a–e Statistically different by Tukey’s post hoc test (p<0.05).

DE, digestible energy; ADCGE, apparent digestibility coefficient of gross energy; DP, digestible protein; ADCCP, apparent digestibility coefficient of crude protein; ADCN, apparent digestibility coefficient of nitrogen; ANOVA, analysis of variance.

## Data Availability

Upon reasonable request, the datasets of this study can be available from the corresponding author.
